# Implications of Visual Attention Phenomena for Models of Preferential Choice

**DOI:** 10.1037/dec0000049

**Published:** 2016-02-01

**Authors:** Timothy L. Mullett, Neil Stewart

**Affiliations:** 1 Psychology Department, University of Warwick

**Keywords:** attention, choice models, drift diffusion, eye-tracking, gaze cascade

## Abstract

We use computational modeling to examine the ability of evidence accumulation models to produce the reaction time (RT) distributions and attentional biases found in behavioral and eye-tracking research. We focus on simulating RTs and attention in binary choice with particular emphasis on whether different models can predict the late onset bias (LOB), commonly found in eye movements during choice (sometimes called the gaze cascade). The first finding is that this bias is predicted by models even when attention is entirely random and independent of the choice process. This shows that the LOB is not evidence of a feedback loop between evidence accumulation and attention. Second, we examine models with a relative evidence decision rule and an absolute evidence rule. In the relative models a decision is made once the difference in evidence accumulated for 2 items reaches a threshold. In the absolute models, a decision is made once 1 item accumulates a certain amount of evidence, independently of how much is accumulated for a competitor. Our core result is simple—the existence of the late onset gaze bias to the option ultimately chosen, together with a positively skewed RT distribution means that the stopping rule must be relative not absolute. A large scale grid search of parameter space shows that absolute threshold models struggle to predict these phenomena even when incorporating evidence decay and assumptions of either mutual inhibition or feedforward inhibition.

When choosing between alternatives, individuals spend varying amounts of time deliberating over their choice. While doing so they shift their attention several times between different items and attributes. Importantly, these shifts in attention can be used to predict choice, and in recent years there has been increasing interest in discovering what attentional shifts can tell us about the underlying decision process (Orquin & Mueller Loose, 2013). The primary tool underlying this advance has been eye tracking and a number of robust phenomena have been identified which link visual attention to the decision making processes. The most robust have been the late onset gaze bias (LOB), sometimes referred to as the gaze cascade (Shimojo, Simion, Shimojo, & Scheier, 2003), and the mere exposure effect (Bird, Lauwereyns, & Crawford, 2012). As a result, models have been formulated with the explicit aim of explaining these phenomena (Krajbich, Armel, & Rangel, 2010; Krajbich, Lu, Camerer, & Rangel, 2012; Shi, Wedel, & Pieters, 2013) and debate has persisted over how well existing models cope with these new findings (Fiedler & Glöckner, 2012; Glöckner & Herbold, 2011; Stewart, Gächter, Noguchi, & Mullett, 2016; Stewart, Hermans, & Matthews, 2015). In this article we aim to avoid discussing the relative merits of specific models and instead focus on more general properties which can inform the taxonomy of evidence accumulation models. Primary among these properties is the stopping rule: the criterion by which a model stops accumulating evidence and makes a response. Specifically, we investigate whether different stopping rules can predict the pattern of visual attention in decision making.

The mere exposure effect has long been noted in Psychology and Marketing (Zajonc, 1968), with consumers generally preferring a product which they have heard of and is familiar to them (Fang, Singh, & Ahluwalia, 2007; Winkielman & Cacioppo, 2001). This is true even if this familiarity is not accompanied by any knowledge about the product which would be relevant to the choice (Mandler, Nakamura, & Van Zandt, 1987; Nordhielm, 2002). Recent studies have shown that the mere exposure effect is evident at much shorter timescales and is evident within deliberation of a single choice (Atalay, Bodur, & Rasolofoarison, 2012). Furthermore, this effect can be used to bias preference. For example, when a display alternates between two different faces, individuals are more likely to prefer the face which was presented for longer (Bird et al., 2012; Shimojo et al., 2003). This is true even when exposure time differs by only a few hundred milliseconds. The majority of studies and models interpret the bias during deliberation as showing that individuals accumulate evidence in favor of choosing an item and that there is a bias toward accumulating evidence more quickly for the item currently attended (Armel, Beaumel, & Rangel, 2008; Kim, Seligman, & Kable, 2012; Krajbich et al., 2012; Mitsuda & Glaholt, 2014; Noguchi & Stewart, 2014; Shimojo et al., 2003; Simion & Shimojo, 2006). Therefore, assuming all items are positive, if one item is attended for longer, more evidence is accumulated in its favor and it is more likely to be chosen. The mere exposure effect is important for our modeling because all the models we implement assume faster accumulation for attended items.

The LOB describes the finding that in the final moments before decision, individuals are far more likely to be looking at the item they are about to choose (Shimojo et al., 2003). When this looking bias is plotted against time it is shown to increase slowly at first, beginning approximately one second before the decision, but then rising rapidly until the moment the response is made, or very slightly before (see Figure 1). Crucially, experiments have shown that this is reflective of the underlying deliberation process (Krajbich et al., 2012; Shimojo et al., 2003; Simion & Shimojo, 2007). The cascade continues even when the visual display turns blank, suggesting that shifts in visual attention reflect covert shifts in the item currently being considered (Simion & Shimojo, 2007). Furthermore, individuals are not merely attending to an object as part of planning the response action, for example, looking left to aid in pressing the left button. Nor are they simply halting visual search while they make the requisite motor response. We know this because the cascade is still present when participants are forced to look away from the pictures in order to make their response (Glaholt & Reingold, 2009) and the final fixation on an item is often shorter than those preceding it, suggesting it is interrupted by a decision threshold being crossed (Krajbich et al., 2010). In addition, the characteristics of the LOB react and change subtly depending on the specific task with the cascade beginning earlier when there are more alternatives and the choice is more complex (Glaholt & Reingold, 2009; Glaholt, Wu, & Reingold, 2010; Schotter, Berry, McKenzie, & Rayner, 2010; Schotter, Gerety, & Rayner, 2012). This is evident in Figure 1, where panel a comes from a simple binary choice experiment, panel b comes from a complex multiattribute decision, and panel c comes from a risky gambles experiment. Although the three plots have differences, the LOB has some stable characteristics. It is characterized by attention being split equally between items in the early and middle portions of the trial, before rising during the last moments before decision and finishing with a bias to be looking at the chosen item 60–90% of the time, depending on choice complexity. Furthermore, although it is not often reported, in our own studies we have found that the LOB onsets at the same point prior to decision, regardless of how much time an individual has spent deliberating the choice beforehand. That is, trials with different RTs show the same absolute duration LOB.[Fig-anchor fig1]

One of the aims of this study is to examine competing explanations for the LOB. One prominent explanation is a feedback loop between attention and evidence accumulation. Individuals are biased to pay more attention to rewarding items. Therefore, as one item begins to be preferred, there is a bias to fixate on it more, which in turn increases the probability of accumulating evidence in its favor (Glöckner & Betsch, 2008; Glöckner & Herbold, 2011; Shimojo et al., 2003). This explains findings that the cascade is larger when individuals are selecting the option they like, as opposed to dislike (Mitsuda & Glaholt, 2014; Simion & Shimojo, 2006). However, others have suggested that the bias in looking can be better explained by the relevance of the evidence to the choice. In “like” tasks, individuals focus more on the positive information whereas in “dislike” tasks they do the opposite (Armel et al., 2008; Meloy & Russo, 2004; Schotter et al., 2010). More recent evidence suggests that early in deliberation attention is biased toward the more rewarding stimuli, regardless of the current task, suggesting a bottom up effect on attention; then, a top-down system exerts more control as deliberation continues, with attention being biased toward the item containing evidence most relevant to the current decision task (Kovach, Sutterer, Rushia, Teriakidis, & Jenison, 2014; Schotter et al., 2012). In the case of reject tasks, attention becomes biased toward negative options and the gaze cascade is still toward the item which is selected as the worst. Given the nuances and complexity of the arguments about whether positive evidence is always accumulated or whether negative evidence is accumulated in reject tasks as well as effects of bottom up and top down control, we focus here on the highly robust effects seen in preferential choices between positive items. Thus we do not address what characteristics may be driving the loop but focus on the more fundamental question of whether any kind of loop is required, or indeed capable, of producing the LOB.

The competing explanation for the cascade effect is that there is no loop, and no exogenous effects on attention (Krajbich & Rangel, 2011), with the exception of ignoring eliminated options (Glaholt & Reingold, 2009; Glaholt et al., 2010). Instead, attention is allocated randomly and the cascade emerges because of a fundamental property of the decision making process. This interpretation posits only that individuals are biased to accumulating evidence in favor of the currently attended item. Each item has its own accumulator, which is incremented as more evidence is acquired and a decision is only made once a threshold is reached thereby satisfying a stopping rule. The LOB is predicted because evidence is accumulated more rapidly for the attended item. Therefore, when the threshold is reached and a response made, it is more likely that it is the currently attended item which has just accumulated the requisite evidence. By plotting attention locked to the point at which a decision is made, one is retrospectively plotting this antecedent shift in attention and the resulting increased accumulation rates which caused the choice to be made.

This article examines whether the LOB can be produced by a model in which attention is not exogenously controlled and is allocated randomly. Furthermore, it investigates which stopping rules can reliably predict a cascade. Most models fall into one of two categories: relative threshold models (Busemeyer & Townsend, 1993; Ratcliff & McKoon, 2008) and absolute threshold models (Usher & McClelland, 2001). For models using a relative threshold, each item has its own accumulator and when evidence is gathered for one item it increments that particular item’s accumulator. A decision is made when one item has accumulated enough evidence to be far enough ahead of the other. That is, a decision is made when the *difference* between accumulators reaches a set threshold. When there are more than two alternatives then one must start making additional assumptions about the relativistic comparison (e.g., highest accumulator vs. 2nd highest, or highest vs. average). This particular debate is beyond the scope of this review because we deliberately focus only on binary decisions (for a discussion see Krajbich & Rangel, 2011; Teodorescu & Usher, 2013).

In absolute threshold models, each item has its own accumulator. However, a decision is made when any one accumulator reaches a set threshold, regardless of the state of the competing accumulator. Arguably there is more variation between the many models which employ an absolute stopping rule. These models often posit differing types of interaction and inhibition between the accumulators or inputs during evidence accumulation (see Teodorescu & Usher, 2013, for a review). We take the most common assumptions and functional forms, then examine their fundamental properties in relation to the visual phenomena we are modeling. Toward the end of the article we also apply a very general approach which aims to identify whether particular assumptions could ever produce these phenomena.

The simplest absolute threshold model examined here is the independent race model, where there is no interaction and the accumulators simply race toward the threshold (Vickers, 1970). Although useful for illustrating simple effects, this model is rarely used in choice fitting as it is poor at predicting RT patterns. The first alternative tested is an assumption of mutual inhibition (Usher & McClelland, 2001). This describes a model where the total evidence accumulated for an option inhibits the input to competing accumulators. As one accumulator gets close to the decision threshold it is inhibiting the accumulation of new evidence for other items, meaning it is able to increase its lead even more quickly. This could be a potential explanation for a LOB. Another possible assumption is that of feedforward inhibition (Krajbich et al., 2010; Mazurek, Roitman, Ditterich, & Shadlen, 2003; Niwa & Ditterich, 2008; Shadlen & Newsome, 1996). In feedforward inhibition, the inputs to each accumulator have an excitatory effect on their own accumulator, but an equal or proportional inhibitory effect on competing accumulators. This is mathematically equivalent to normalizing the values at the input stage. Interestingly for this study, in binary choices a model with full inhibition is mathematically equivalent to a relative threshold model. The relativity is simply implemented at the evidence accumulation stage rather than the decision threshold stage: With complete inhibition, differences are accumulated directly. Therefore the simple relativistic rule implemented here can also be represented as a special case of the feed-forward absolute threshold rule, where inhibition is 1 and decay is 0.

There are several sections to this article, each with its own purpose. To begin we use simple toy models whereby each simulated fixation results in a one unit increment of evidence. These simplified models are included to more clearly demonstrate the effects of differing assumptions. In the first section we demonstrate that a simple absolute model is unable to produce the LOB and demonstrate the reason for this. We then show that a very simple relative model is able to produce a LOB even when attention allocation is entirely random. The second section shows that the results remain the same when there is only a probabilistic link between attention and evidence accumulation. In the third section, we demonstrate the effect of adding a feedback loop as suggested by several visual attention models, which actually makes the model predictions worse. The fourth section addresses different inhibition assumptions within the absolute threshold model. It is shown that a simple mutual inhibition model cannot predict the LOB and that the feedforward model can produce similar results to the relative threshold model, but only when inhibition is near 100%.

Finally, we present a large scale grid search of parameter space for a relative threshold model, a mutual inhibition model, and a feedforward inhibition model (see Table 1). The relative threshold model is able to predict simultaneously the qualitative characteristics of the LOB and many common properties of measured RT distributions with a single set of parameters. For the mutual inhibition model, we find parameter values which can predict the LOB but also result in unrealistic RT distributions and we find values which can predict RT distributions, but fail to produce the LOB. There is no parameter set which can simultaneously predict both. We find similar results for the feedforward inhibition model, with no single set of parameter values which can predict the LOB and RT distributions simultaneously. This model in particular we find to be very sensitive to small changes in parameter values, especially the level of inhibition.[Table-anchor tbl1]

## Simulations

### Absolute Threshold Models

The first model reported is that using a simple absolute threshold with attention having a deterministic effect on evidence accumulation. This simple absolute stopping model is no longer a serious candidate in the literature. Current absolute stopping models also incorporate inhibition between accumulators or inputs or both, and decay or leakage, and trial-to-trial variation in parameters. These additions are necessary to capture key results in choice and RT data. We start with this model, however, to illustrate the basic problem in producing a LOB. Below we show that incorporating the additional mechanisms listed above does not allow the models to capture simultaneously the LOB and even just basic properties of the RT distribution.

**Figure 2 fig2:**
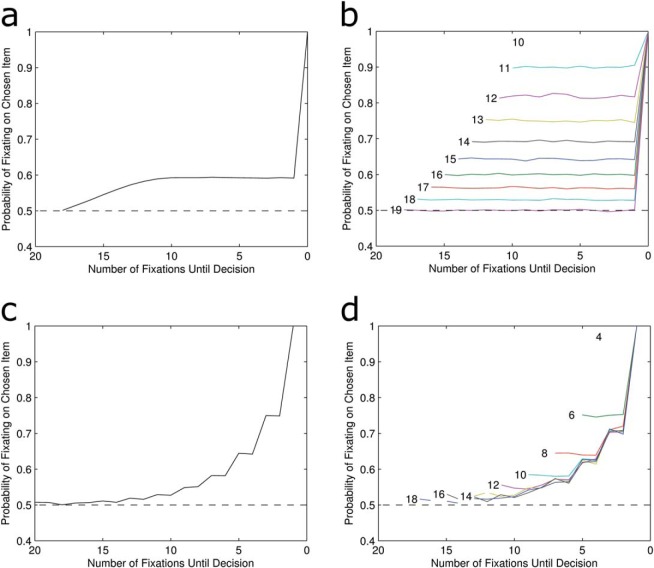
Average LOB for absolute threshold model (a) and relative threshold model (c). Also, shown are the patterns of LOB broken down by length of deliberation time (RTs) for absolute (b) and relative (d). See the online article for the color version of this figure.

The model assumes that for each fixation, the attended item has its accumulator incremented by 1. To set the threshold we used a grid search to find the parameter that gives a RT closest to 16 fixations. We simulated 100,000 choices and the LOB was plotted by calculating the proportion of simulated choices where the preferred item was fixated at each time point, working back from the moment the decision was made. Figure 2a shows that this model fails to produce the classic LOB. Instead of a cascade there was a bias toward the chosen item which appears to develop gradually from the beginning of the choice, then plateaus early on. There is then a sudden tick up for the very last fixation, with 100% of trials ending while the preferred item is fixated. The latter is easily explained: because evidence can only be accumulated for the fixated item the accumulator can only be incremented to cross the decision threshold while the ultimately chosen item is being attended.[Fig-anchor fig2]

The cause of the long-term bias throughout the choice is less obvious. To see why this occurs it is helpful to plot the pattern of the LOB for choices with different RTs (Figure 2b). This reveals that for any choice with a given RT, the probability of having fixated on the chosen item is constant throughout. Consider a trial where a response is made after only 10 pieces of information have been collected. In this case there were exactly 10 fixations and since an accumulator has reached the decision threshold all 10 must necessarily have been toward that one item. Therefore there was a 100% chance for each fixation to have been directed to the chosen item. For a choice where exactly 11 pieces of evidence were accumulated, one knows that 10 of these must have been directed toward the chosen item. One also knows that, because of the deterministic nature of the model, the final fixation must have been directed toward the chosen item. Therefore, for the first 10 fixations there is a 90% chance that an individual fixation was toward the chosen item. However, what is important for the failure to produce a LOB is that the order in which the evidence was accumulated is irrelevant to the choice. It is not specified exactly when within the first 10 fixations attention was directed to the now chosen option. Only the last fixation is determined, hence the plateau followed by the sudden tick to 1.

Figure 2b also makes clear that the reason for the gradual rise in gaze bias at the start of the trial is simply because of averaging over different lengths of trials. Trials with longer RTs necessarily involve more fixations toward the nonchosen item and are included in the averaged LOB before the shorter RT trials have even begun. As one moves closer to the decision point, trials with short RTs and much larger biases begin to be included in the average. This is why the bias plateaus 10 fixations before the decision.

### Relative Threshold Models

Now we turn to models which assume that decisions are made using a relative stopping rule: instead of stopping when either item reaches a set threshold, the decision depends on the size of the difference between evidence accumulated for either item. As this model uses a different decision rule, one cannot use the same threshold value as in the absolute threshold—after all a value of 5 for the threshold, for example, would be the difference in evidence for a relative stopping rule but would be the total evidence for one accumulator in an absolute stopping rule. A grid search was again used to identify a difference threshold of 4 as providing a mean RT closest to 16. The model simulated 100,000 choices and the LOB was plotted by averaging attention over all choices. Figure 2c shows that the pattern matches the classic LOB finding. The probability of fixating the chosen item increases with a convex shape just prior to the decision being made.

The LOB was then plotted for trials of different RTs. Unlike the absolute threshold rule, the relative rule could potentially produce infinitely long RTs as a result of random walk dynamics. However, the probability of observing longer RTs drops quickly and they are rare in these simulations. To allow for this we only plot discrete RTs which occurred in at least 5% of simulated choices.

Figure 2d shows that the LOB develops with a very similar time course for all RTs. There was a slight early bias evident in very short trials which deviates from the average cascade. However, this was much too small to be driving the overall effect and quickly converges to match the fixation biases of other RTs. Crucially, a pattern of increasing bias was found as the decision point approaches, regardless of the trial’s RT. This is because creating a relative difference between the accumulators requires a sequential series of fixations on one item and a decision will be reached at the end of this run. So unlike the absolute threshold rule, there is a constraint on the order in which evidence is accumulated leading up to a response.

### Nondeterministic Evidence Accumulation

**Figure 3 fig3:**
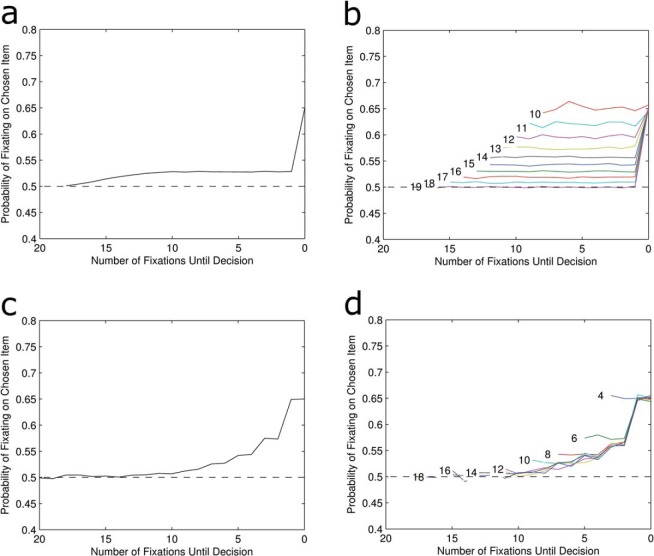
Average LOB for absolute threshold model (a) and relative threshold model (c) when attention is modeled as having a probabilistic bias on evidence accumulation. The results show no qualitative differences compared to the deterministic rule, even when the patterns of LOB are separated by RTs for both absolute (b) and relative (d). Note the change in scale from Figure 2. See the online article for the color version of this figure.

One may think it possible that these results are attributable to the improbable assumption of a deterministic rule linking fixations to evidence accumulation. Indeed models of evidence accumulation generally predict only a probabilistic bias toward accumulating evidence for the attended item (Krajbich et al., 2010). To address this, both the absolute and relative threshold models were run again, but this time fixating on an item resulted in a 65% chance of incrementing the accumulator for the attended item. In the other 35% of cases, evidence was accumulated for the unattended item instead. The bias of 65% was chosen to approximately match the bias that attention has on evidence accumulation as has been estimated in empirical studies (Krajbich et al., 2010). Figure 3 shows that the pattern of results is virtually identical. The only difference for the absolute threshold model is that the size of the early bias has been reduced and the final tick only represents 65% of trials where the final fixation is on the chosen item. The probabilistic rule means that the overall bias effect cannot be more than 65% but it does not change the underlying reason for the lack of LOB: the order in which evidence is accumulated does not matter in the absolute threshold model. For the relative threshold, the results still show a cascade of increasing bias toward the chosen item before decision but now the cascade has a maximum bias of 65%.[Fig-anchor fig3]

### Biases in Attention Allocation

One common interpretation of the LOB is that attention is biased toward the currently favored item, creating a positive feedback loop. Essentially, as more evidence is accumulated for an item it is more likely to be attended, thus producing the LOB in the final moments before choice when evidence is highest. This is interesting as it is intuitively plausible that an absolute threshold rule could produce the LOB when combined with attentional bias. Therefore the modeling approach is applied to test this intuition. The only difference from the absolute threshold model described in the above model is in the allocation of attention. In the previous model this was entirely random and independent of previous fixation locations. In this model the following equation was used:

$${\text{ProbAtten}}_{left}=\frac{E_{left}+b}{E_{left}+b+E_{right}+b}$$ 1

where *E* is the total evidence accumulated for the left or right items and *b* is a baseline constant added to ensure that small early biases do not overwhelm the equation and dictate all subsequent fixations. Within the simulation, *ProbAtten* is compared with a number between 0 and 1 randomly drawn from a uniform distribution. If *ProbAtten* is greater than this number then Attention is directed toward the left item, otherwise it is directed toward the right. The value of *b* = 3 was used to produce the reported results, however there is no qualitative change when b is given very high values.

**Figure 4 fig4:**
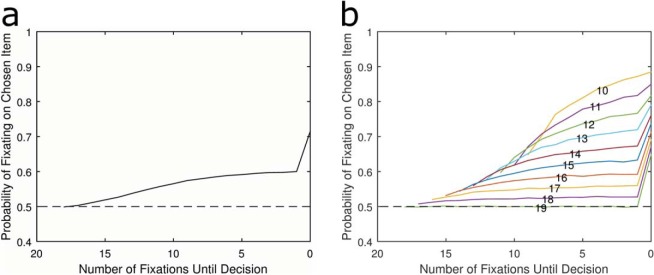
The LOB is plotted for the absolute threshold rule when attention is exogenously biased toward the currently preferred item. See the online article for the color version of this figure.

Figure 4 shows that even with a substantial attention bias, the absolute threshold decision model is not able to produce the LOB. When attention bias is plotted separately for different RTs, there is a clear increase in the probability of attending the chosen item. However, this occurs early on and the effect is still very dependent on the RT itself. Where there is a long RT, the fixations must necessarily have been split relatively equally between the items. Therefore there was never a large difference between the evidence accumulated, meaning there was also never a large attention bias toward either.[Fig-anchor fig4]

### Inhibition

A number of accumulator models incorporate inhibition, whereby the rate of evidence accumulation for any option is directly affected by the other items available. This is especially relevant here because depending on the specific implementation of inhibition, these models can be mathematically identical to relative threshold models when predicting choice and RTs. That is, when modeling choice and RTs in a two alternative decision, they will make identical predictions. In this section we explore how attention effects can differentiate between them.

A number of models, including leaky competing accumulators (Usher & McClelland, 2001), assume the inhibition of evidence accumulation is dependent on the total evidence accumulated for the competing option. That is, if one option has accumulated so much evidence that it is close to reaching the decision threshold, it will almost entirely inhibit the accumulation of evidence for the competing option. To simulate this pattern of accumulation, attention is randomly allocated on each time step. When attention is directed at item 1 we implement evidence accumulation using:

$$\begin{array}{l} \Delta E_{1}=AV_{1}-\frac{\left(1-A\right)IE_{2}}{T}\\ \Delta E_{2}=\left(1-A\right)V_{2}-\frac{AIE_{1}}{T} \end{array}$$ 2

where Δ*E*_*i*_ is the change in accumulated evidence for item i, *E*_*i*_ is the total evidence accumulated, and *V* is the value input from the stimulus. *A* is the attention bias toward accumulating evidence for the attended item, *I* is the inhibition parameter, and *T* is the decision threshold. This normalization by the threshold means that if *I* = 1, when one item has accumulated enough evidence to be half way to the decision threshold, the input to the competing item is reduced by 50%. As the simulation uses the same absolute threshold rule as the previous models the same parameters are applied. To demonstrate the qualitative effect of inhibition most clearly, the parameter *I* is set to 1.

**Figure 5 fig5:**
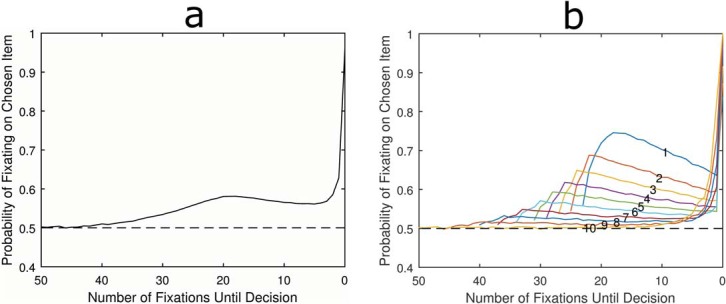
Average LOB for absolute threshold model with mutual inhibition (a) and the LOBs from the same model when simulations are split into deciles by RT. See the online article for the color version of this figure.

Figure 5a shows that the resulting LOB shows a very early rise from chance, followed by a decline before a rapid rise for the last few fixations. The reason for this pattern becomes clear when the plots are split by RT. Figure 5b shows the gaze cascades when responses are plotted separately for each decile of RT. For the quickest responses, there is a very rapid early rise, then a distinct decline. This is because for a short RT, early fixations must be significantly biased toward one item. This item therefore accumulates a lot of evidence and rapidly begins to inhibit accumulation for its competitor. When the difference between them becomes large, the inhibition will be so great that attention bias is largely irrelevant. This is why the attention bias then declines closer to chance. In the case of longer RT trials, fixations are split evenly between the two options early on, meaning mutual inhibition builds and it takes a run of quite a few fixations to one item to break the balance. This is the reason for the earlier onset of the final increase in bias in longer RT trials.[Fig-anchor fig5]

We also report simulation results for feedforward, or input inhibition. This refers to a model where the value input from a stimulus inhibits the value input from the competing item:

$$\begin{array}{l} \Delta E_{1}=AV_{1}-\left(1-A\right)IV_{2}\\ \Delta E_{2}=\left(1-A\right)V_{2}-AIV_{1} \end{array}$$ 3

This model has the interesting property that when inhibition is equal to 1, it becomes mathematically equivalent to a relative threshold model without inhibition. However, the relativity enters the model at the point of evidence accumulation, instead of later, at the decision rule. Our first aim here is to establish whether both assumptions are able to predict the LOB. Because of this functional similarity, the same threshold value of 4 was initially used, however due to the inhibition term reducing the amount of evidence accumulated on each fixation RTs were implausibly long. A threshold of 2 is instead used, though the qualitative results remain the same.

**Figure 6 fig6:**
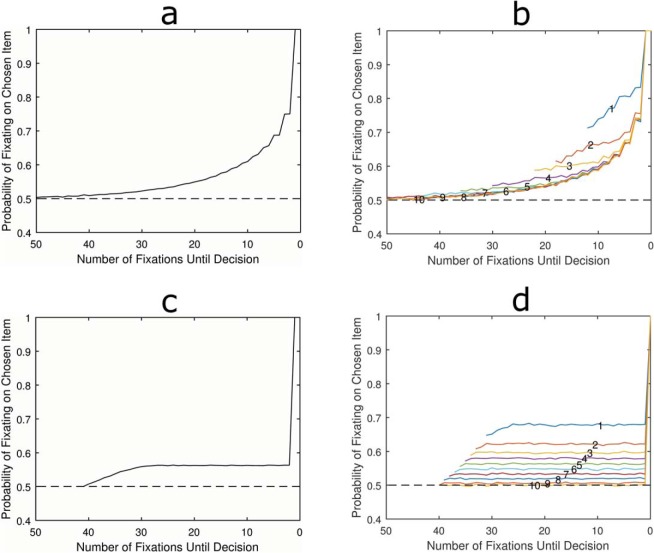
The average LOB for an absolute threshold rule with feedforward inhibition when *I* = 1 (a) and when *I* = 0.5 (c). This shows that when inhibition is high, such a model can produce the LOB, and mimic the relative threshold rule. In addition, when split into deciles by RT, the model with full inhibition shows gradual increase in bias across all trial lengths (b), whereas when *I* = 0.5, the bias is flat within each RT decile (d). See the online article for the color version of this figure.

Figure 6a shows that when inhibition is set to 1, the model produces the desired pattern of a monotonically rising bias immediately before choice. Figure 6b shows that some of this is caused or accentuated by shorter RT trials having a higher bias overall. However, this effect is very different to that found in other absolute threshold models because in all cases the bias increases over the final few fixations rather than being flat and then showing a sudden spike.[Fig-anchor fig6]

Next we examine what happens to the LOB when inhibition is not equal to one, thus breaking the equivalence to relative threshold models. The reason for this is that empirical fitting of behavioral data rarely yields such high estimates of inhibition. Therefore, an additional simulation was performed with inhibition reduced to 0.5. Figure 6c shows that the gaze cascade now looks nearly identical to that produced by the corresponding model with no inhibition at all.

**Figure B1 fig18:**
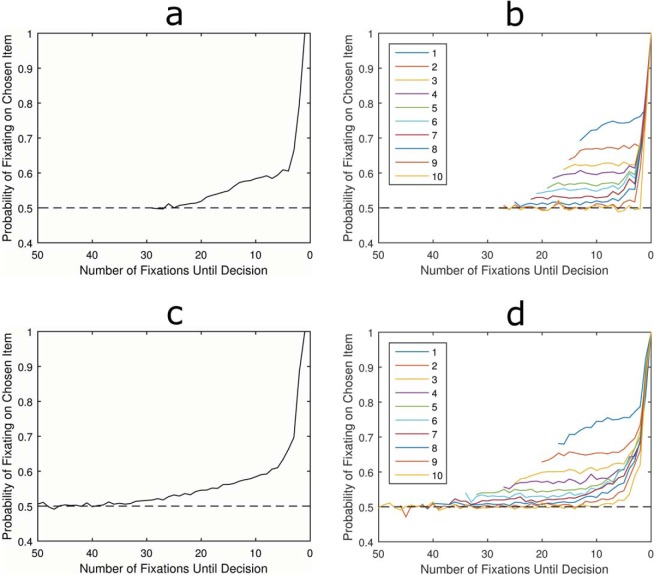
This shows the LOB for an input inhibition model. Plot a shows the average LOB for a value of I = 0.8 and plot b shows the LOBs separated by decile of reaction times. Plots c and d show the same for a model where I = 0.9. See the online article for the color version of this figure.

The reason for this large change in the LOB with partial rather than complete inhibition is as follows. With complete inhibition (*I* = 1), the attended and unattended items have their accumulators increased and decreased respectively by the same amount (in this simulation, by + 0.3 and −0.3). This is analogous to a single item accumulating 0.6 in a relative threshold rule because the total evidence accumulated at any time is zero. However, with partial inhibition (*I* = 0.5) the attended item is increased by 0.48 and the unattended is also increased but only by 0.03. Therefore, the model is no longer equivalent to that of the relative threshold and because the gain of attending an item is greater than the penalty of attending its competitor, the resulting LOB is similar to that of other absolute threshold models. The plateau pattern is still evident even when *I* is large enough for the unattended item to experience negative accumulation, just so long as this is smaller than the positive gain associated with being attended. In fact Figure B1 in the Appendix B shows that even when I = 0.9 there is notable flattening in the LOB for separate RT trials and when I = 0.8 the plateau pattern is obvious in the overall LOB.

### Real Time Simulation and Model Tests

The simplified assumption of each fixation resulting in a single quantum of evidence being accumulated is useful for illustrating the effects of differing RTs. However, the majority of drift diffusion models posit that evidence is accumulated continuously over time and that the accumulation of evidence at each timepoint is biased toward the item which happens to be currently fixated (Busemeyer & Townsend, 1993; Krajbich et al., 2010; Krajbich & Rangel, 2011). Furthermore, in the models presented up until this point we have deliberately manipulated as few parameters as possible so that we can more easily identify the effect of individual model properties. However, nearly all models of choice are fitted with several estimated parameters, all of which can interact and may combine to produce the LOB and RT patterns common in choice data. The following modeling addresses both of these issues. First, we assume that evidence is accumulated constantly and over a series of fixations with varying durations. Second, we perform a large scale grid search of the parameter space to test what proportion of the potential parameters can predict properties of the LOB and predict RT distributions that match those commonly found in empirical studies.

**Figure A1 fig17:**
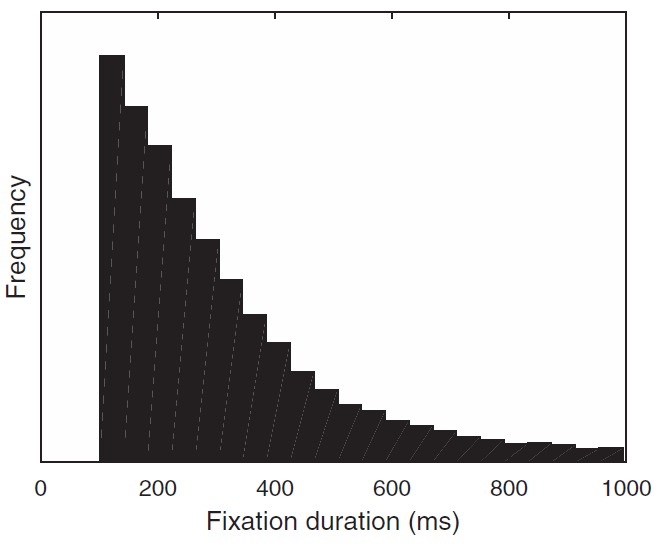
A histogram showing the distribution of fixation durations measured in Mullett and Tunney (2016) and sampled from during real-time simulations.

To approximate continuous evidence accumulation the time during deliberation was split into bins of 10ms. At the start of each choice, a fixation duration was sampled from the distribution measured in Mullett and Tunney (2016; See Figure A1 in Appendix A for the distribution of sampled fixation durations). Attention was randomly assigned to one of the items and evidence was sampled with an attentional bias for the duration of the randomly sampled fixation. At the end of this fixation a new duration was sampled and attention was again randomly assigned.

We begin with a relative threshold model. The parameters that are allowed to vary are the attention bias, *A*, and the decision threshold, *T*. The minimum value for *A* is set at 0.5, as this value results in no bias to either option. A value of less than 0.5 would lead to a bias toward the unattended item. Thirty potential values of *A* were examined, each of which were spaced linearly between 0.5 and 1. For the threshold value there is no upper bound therefore a maximum was selected that would explore the extremes of the model’s range. We selected a minimum of 2 and a maximum of 100. Again, 30 values were selected, equally spaced between. This resulted in 900 combinations, each of which were simulated for 33,000 choices. (33,000 choices is the maximum given the large computer memory burden of storing the entire sequence for every simulation.) To enable computational tractability and keep these simulations within the capacity of our equipment, the simulation would terminate once the simulated RT was more than 30 seconds. If a decision had not been reached then a null response was recorded.

The large number of parameter combinations means it is not possible to discuss them individually. Therefore we implement a number of qualitative checks on the predicted LOBs and RT distributions. These enable us to specify characteristics which are present in individuals’ behavior and then objectively test which parameter sets and which models satisfy them. These can then be used to partition the parameter space and report the volume of parameter space in which our criteria are met (Lee & Corlett, 2003; Pitt, Kim, Navarro, & Myung, 2006). In total we use five criteria listed in Table 2.[Table-anchor tbl2]

**Figure 7 fig7:**
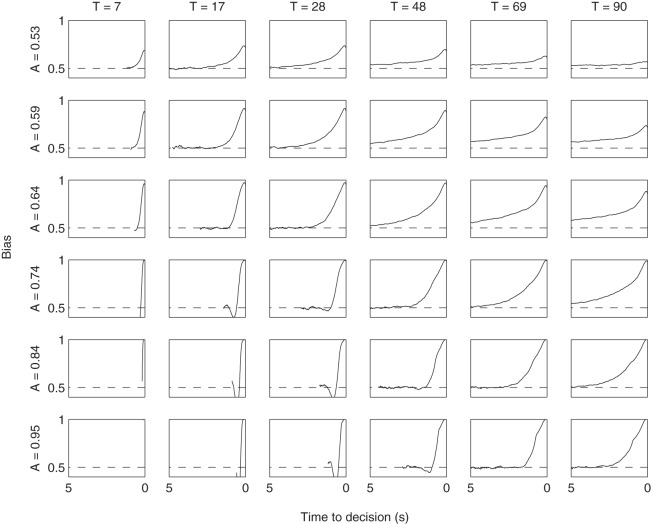
The LOB plots estimated by the grid search of parameters for the relative threshold rule. Columns list different values for the threshold *T*, and rows correspond to values of attention bias *A.*

**Figure 8 fig8:**
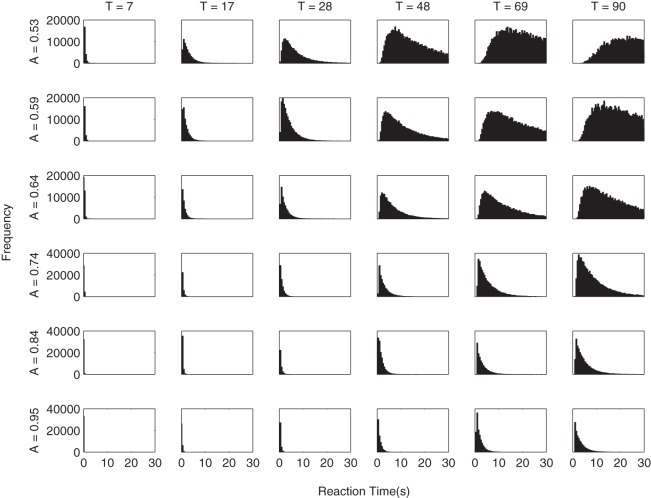
The RT distributions estimated by the grid search of parameters for the relative threshold rule. Columns list different values for the threshold *T*, and rows correspond to values of attention bias *A*.

Simulating the relative threshold model shows that there are parameter combinations which satisfy all five of these criteria simultaneously (see the first column of Table 3). These are in a small minority, but the fact that these values are found demonstrates that the underlying properties of this two parameter relative threshold model allow it to simultaneously fit the behavior observed in visual attention and choice experiments. Figure 7 shows the gaze cascades produced by a small but representative sample of the parameter space, and Figure 8 shows the corresponding distributions of RTs. The observable effects are all quite intuitive. A higher threshold means that responses take longer and the gaze cascade becomes more stretched over time as a longer run of samples is required for an item to reach the higher threshold. Increasing the attention bias serves to shorten RTs as each increment of information has a larger effect. It also increases the magnitude of the bias, but not in a linear manner because of the properties of a biased random walk.[Table-anchor tbl3][Fig-anchor fig7][Fig-anchor fig8]

### Absolute Threshold and Mutual Inhibition

We now turn to models with an absolute stopping rule, beginning with one that implements mutual inhibition. For these absolute threshold models we allow more free parameters to vary and test a very large number of combinations. This added complexity allows us to better represent the general properties of absolute threshold models commonly fitted to behavioral data. In total we vary four parameters: attention bias *A*, the decision threshold *T*, the decay rate *d*, and the inhibition parameter *I*. The decay rate is implemented at the beginning of each time point using:

$$E_{t}=E_{t-1}\left(\frac{1-d}{t}\right)$$ 4

This equation means that when *d* = 1 evidence decays at a rate equal to the maximum rate which it can be accumulated. When *d* = 0, there is no decay at all and perfect responding would be possible, given enough time. Once the decay rate has been applied, evidence accumulation is calculated using Equation 2.

The parameters *d* and *I* are all bounded by 0 and 1. Ten equally spaced values were tested, with these bounds used as the maximum and minimum values. For *A*, we used the same bounds as in the relative threshold model and tested 10 equally spaced values between. The parameter *T* again has no upper bound and as demonstrated earlier the threshold in an absolute model must generally be higher than in the relative threshold model, meaning a large value could be plausible. Simultaneously, the interaction with decay and inhibition will mean that for certain parameter configurations a very low threshold could be plausible. To cover these extremes we always included very small values of *T*: 1, 3, and 5. We then set our maximum to 320 and selected 10 evenly spaced values between 10 and 320. This resulted in 13,000 combinations of parameter values and, as in the relative threshold model, 33,000 choices were simulated for each.

As the second column in Table 3 shows, none of the 13,000 tested combinations were able to simultaneously satisfy all 5 criteria, though a small number were able to satisfy 4. It is not possible to simultaneously show the effect of varying all 4 parameters so instead we present a series of plots for each parameter. The values of three out of four parameters are kept constant and the plots show the cascade and RT distribution for each tested level of the remaining one. The values of the remaining three parameters are selected to present results that perform well, but their primary purpose is to demonstrate the effect of varying the parameter of interest. In all cases we seek to present the clearest possible demonstration of the effect of changing the parameter of interest.

**Figure 9 fig9:**
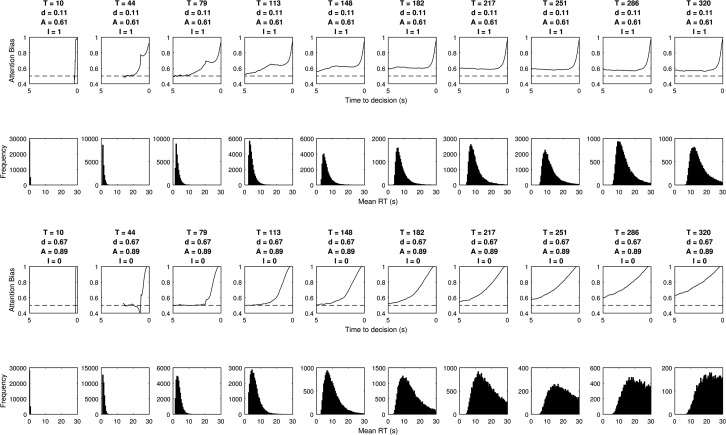
The top row of panels shows the average LOB produced by increasing the threshold parameter in a model with full mutual inhibition. The second row shows the RT distributions for the same models. The lower two rows show the effect of increasing the threshold parameter when inhibition is set to zero.

In Figure 9 the top two rows show the effect of changing the threshold when *I* = 1 and the bottom two rows show the same effect when *I* = 0. Where *I* = 1 we see the same pattern as in the simple example of Figure 5a, where the cascade initially rises and then falls, before a tick up at the end. This increase at the end matches many properties of the LOB, but the model fails because of the large bias preceding it. The threshold increase stretches this out over time, but it is always present. When *I* = 0 the model can perform reasonably well by incorporating decay. This produces good RT distributions and a rise in the final moments, without a preceding bias. However, the model fails because the scale of the decay rate required to produce the RT distribution results in 100% of attention being directed toward the chosen item. In many cases, it reaches 100% before the end. In addition the increasing threshold stretches the LOB to form a gradual rise.[Fig-anchor fig9]

**Figure 10 fig10:**
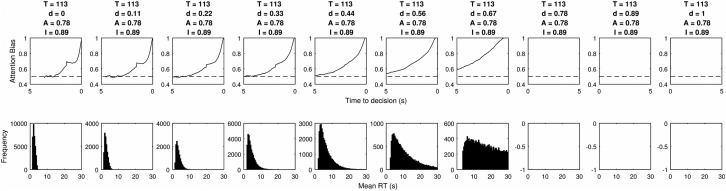
The LOB and RT distributions produced by different levels of decay within a mutual inhibition model. Blank subplots occur for parameter values where too few decisions were made within the imposed deadline of 30s and therefore results cannot be plotted.

In Figure 10 decay is varied. When decay is 0, RTs are all very short and the LOB shows an early rise, then plateau and final sudden tick up. This becomes smoothed as decay is increased, but at the same time the duration of the LOB is extended and onsets earlier than is seen in eyetracking evidence. In addition, 100% of attention must be allocated to the chosen item for some time before choice so that evidence accumulation overcomes the rate of decay. For very high levels of decay the model fails to reach threshold in nearly all choices meaning results cannot be plotted.[Fig-anchor fig10]

**Figure 11 fig11:**
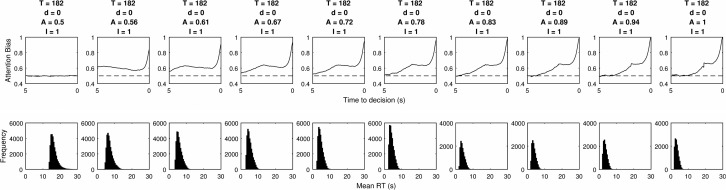
The LOB and RT distributions produced by different levels of attention bias within a mutual inhibition model.

Figure 11 shows that increasing the size of the attention bias while keeping all other parameters constant reduces RT time as the difference in evidence accumulated for either item at each step is larger. It also increases the size of the bias at the point where a decision is made.[Fig-anchor fig11]

**Figure 12 fig12:**
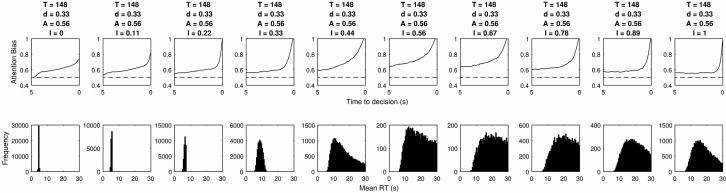
The LOB and RT distributions produced by different levels of inhibition within a mutual inhibition model.

Figure 12 displays the effect of increasing inhibition. This serves to increase RT and allows the model to produce positively skewed distributions. However, an early bias toward attending the chosen item is seen at all levels of *I*.[Fig-anchor fig12]

### Absolute Threshold and Feedforward Inhibition

When fitting an absolute threshold model with feedforward inhibition we use the same parameter values as used for fitting the mutual inhibition model. Evidence decay was also implemented in the same manner. However, evidence accumulation was estimated using equation 3. Again, Table 3 shows that no parameter values were found which satisfied all 5 criteria. Table 4 shows that the criteria the model particularly struggles with is the size of the bias in the final moments. To show the reasons for this we plot the effect of changing each parameter value as was done for the mutual inhibition model.[Table-anchor tbl4]

**Figure 13 fig13:**
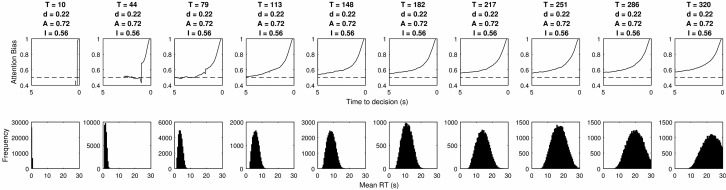
The LOB and RT distributions produced by different thresholds within a feedforward inhibition model.

Figure 13 shows that increasing the threshold increases the average RT, but when the model contains both decay and inhibition the distribution of RTs does not become more positively skewed. The LOB also becomes more elongated, but even with relatively high inhibition and some decay, the model still produces an early bias toward the chosen item.[Fig-anchor fig13]

**Figure 14 fig14:**
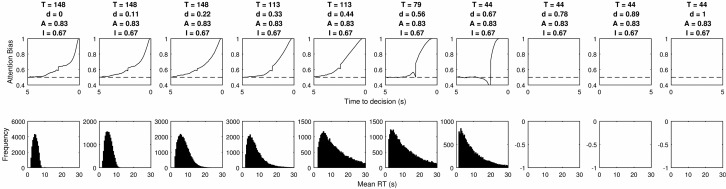
The LOB and RT distributions produced by different rates of decay within a feedforward inhibition model. Blank subplots occur for parameter values where too few decisions were made within the imposed deadline of 30s and therefore results cannot be plotted.

Figure 14 shows that increasing the decay has a large and pronounced effect. At higher levels the decay interacts with inhibition so that no choices are completed, even at low thresholds. High decay rates can also result in attention becoming biased toward the competing item before a sudden upward tick marking the onset of the LOB. This is because the high decay rate combines with the inhibition to mean there is an almost lexicographic and deterministic rule of a decision being made after *X* samples in a row. Thus for a choice to take longer than that number of samples attention must necessarily have been directed to the other item immediately prior to that run. At lower levels of decay the LOB onsets early and results in a gradual rise as well as showing a slight plateau at very low levels, even though inhibition is relatively high.[Fig-anchor fig14]

**Figure 15 fig15:**
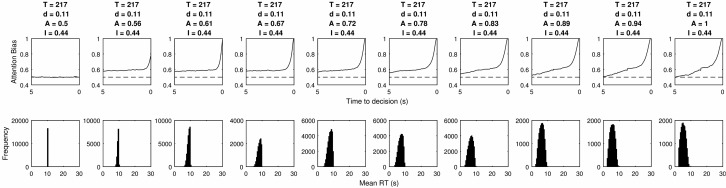
The LOB and RT distributions produced by different levels of attention bias within a feedforward inhibition model.

When attention bias is low, RTs are all relatively similar because the random allocation of attention is introducing less variability into the model. Figure 15 shows that even with a midrange value for inhibition there is an early bias and no positive skew in RTs. At higher levels the relationship becomes more deterministic. Although the LOB onsets much closer to the end of the choice there is still slight early bias and the bias at the time of choice is 100%.[Fig-anchor fig15]

**Figure 16 fig16:**
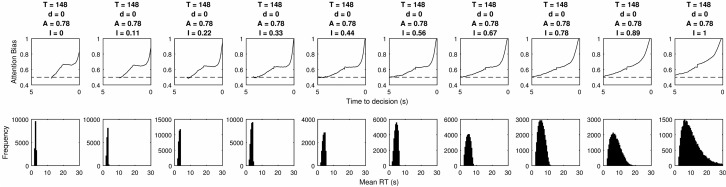
The LOB and RT distributions produced by different inhibition parameter values within a feedforward inhibition model.

Figure 16 shows that increasing the inhibition parameter improves the model’s ability to produce positively skewed RTs with distributions which match behavioral data. However, this also increases the size of the attention bias at the final moment before choice and the resulting predictions are much more extreme than is observed in subjects’ responses. Overall these results show that although there is likely to be a parameter set where the model can match all of these values, the region of parameter space where inhibition, decay and attention bias are balanced is very small. The variance in parameter values estimated from existing choice and RT data is larger by at least an order of magnitude.[Fig-anchor fig16]

## Discussion

Simulations of choice times and attention allocation from evidence accumulation models with relative and absolute stopping rules have revealed two clear results. First, the simulations show that it is possible for a model of decision making to predict the LOB effect without any modulation of attention or feedback loop. Such a loop may still exist, but clearly it is not necessary and in some models including it can actually worsen the model’s performance. Second, we show that an evidence accumulation model with a relative stopping rule can predict the qualitative patterns of the LOB and RT distributions commonly found in empirical data. It does so even when implemented in the most simple form possible, with no decay or inhibition. Models with an absolute stopping rule struggle to explain these effects even with additional assumptions and free parameters. The results of simple simulations show that a model with mutual inhibition fails to produce the LOB, whereas feedforward inhibition can only do so when the inhibition is near 1, something not observed in fits to choice and RT data. A large scale grid search simulating real-time evidence accumulation shows that a relative threshold rule can satisfy qualitative tests for the LOB and RT distributions with a single set of parameters. Conversely we find no parameter sets which can do so for the absolute threshold model with either mutual or feedforward inhibition. The manner in which multiple parameters interact with each other suggests that a set of values does exist for feedforward inhibition given a grid search with very high resolution, particularly when one considers that the relative threshold rule is held as a special case within the absolute threshold model with feed-forward inhibition. However, the area of parameter space is small; much smaller than the variance generally reported when fitting parameters to behavioral data alone.

### Collapsing or Stationary Boundaries

One possibility that we do not directly test is that of collapsing boundaries. This assumption is included in a number of relative threshold models because when evidence for each alternative is equal, then relative threshold rules can predict unrealistically long RTs or fail to converge on a choice at all. Collapsing boundaries address this problem. We do not examine this subclass of models here because we show that even the simplest relative threshold model produces a robust LOB before this additional assumption is included. What is particularly interesting though is that a collapsing boundary can make a relative threshold rule equivalent to an absolute threshold rule when predicting choice and RT (Zhang, Lee, Vandekerckhove, Maris, & Wagenmakers, 2014). This does not qualitatively change our conclusions, as it of course does not affect the predictions of the absolute threshold model, while it simply adds more degrees of freedom to the relative threshold model. However, it means the LOB could be used to test for the presence of collapsing boundaries in future work: a relative threshold rule with collapsing boundaries would predict a different pattern of LOB at different RTs. This is attributable to changes in the relative impact of the accumulation process (which is weighted by attention), and the rate at which the boundary collapses (which is attention agnostic) as deliberation time increases. This effect can also be used in future development of such models to estimate the rate at which a boundary may collapse while simultaneously estimating, and controlling for, the biasing effect of attention.

### Feedback Loop or Patterns Emerging From Random Attention?

The reason that the relative threshold produces the LOB is that a decision is made after several consecutive incidences of evidence being accumulated for one item. By time locking the analysis to the point of response, it is necessarily true that evidence must have been accumulated for the chosen item in the immediately preceding moments. If attention biases evidence accumulation, it must also be true that attention was more likely to be directed toward the chosen item in those immediately preceding moments. The apparent build-up of a bias immediately before a choice gives a powerful intuitive sense of causality: that a deliberate, nonrandom shift in attention causes the resulting decision. However, the results here demonstrate that because the analysis of attention is essentially retrospective, a truly random assignment of attention still predicts the LOB. In fact, assuming a direct loop between accumulated evidence and attention does not alter the models’ predictions. These results are consistent with many recent findings which do not require or assume any systematic bias in attention (Glaholt & Reingold, 2009; Glaholt et al., 2010; Krajbich et al., 2010, 2012; Krajbich & Rangel, 2011; Mitsuda & Glaholt, 2014). They also support studies showing that the mere exposure effect does not require any volitional allocation of attention, and that it is possible for it to be controlled entirely by the environment and presentation duration of stimuli (Bird et al., 2012).

It seems unlikely that attention is entirely random, and several patterns have already been identified (Shi et al., 2013). These include the ignoring of poor options, seemingly because they hit a lower boundary of evidence (Glaholt & Reingold, 2009) and modifying attention switching patterns between items and attributes depending on the stage of choice process (Shi et al., 2013). However, these effects would not be relevant in binary choice. An effect which could present an interesting alternative explanation is that rather than attention being directed more frequently toward the preferred item, attention lingers for longer every time a high value item is attended. Analysis of fixation and dwell durations has indeed shown that they are longer for preferred items, however this does not increase over the duration of the trial (Glaholt & Reingold, 2009, 2011; Glaholt et al., 2010; Orquin & Mueller Loose, 2013). If anything, the effect is most pronounced in the first fixation and reduced during the rest of the trial. Therefore without an increase over time it cannot explain the LOB.

### Stopping Rules and Inhibition in Evidence Accumulation Models

Evidence accumulation models using both relative stopping rules and absolute stopping rules have been shown to fit RT and choice data well in different scenarios (Teodorescu & Usher, 2013). Some studies have also shown that an attention weighted relative threshold model can produce the LOB having been fitted to empirical data based only on RTs and choices (Krajbich et al., 2010). However, this is the first attempt to model both relative threshold rules and absolute threshold rules and examine their fit to behavioral and visual phenomena simultaneously. The results of our grid search analyses show that the relative threshold rule can produce familiar RT distributions and LOB plots which match those from empirical data. It is perhaps surprising that it is such a small proportion of the parameter space which can satisfy all of our qualitative tests simultaneously. However, as Figure 8 shows, the RT distribution is always positively skewed and the LOB always shows a convex rise from a neutral early bias. The fact that the model can capture these qualitative patterns with nearly any viable parameter values shows that a more complex version incorporating inhibition, decay, or collapsing thresholds would find suitable parameter values with even more flexibility to fit behavioral data. In fact prior fits to eye tracking data have used additional assumptions about inhibition (Krajbich et al., 2010).

For absolute threshold models both decay and inhibition were allowed to vary. This is because these parameters are crucial to the majority of absolute threshold models when fitting RT and choice data. Allowing only the threshold and attention bias to vary would result in us only testing the independent race model, which is now included as a special case when decay and inhibition are both 0. Within the literature on evidence accumulation models there is debate about the point at which inhibition should enter the model, either as mutual inhibition of accumulated evidence or as accumulators inhibiting the input to other accumulators in feed-forward inhibition. We use simple simulations to show that a mutual inhibition assumption, based on the total evidence accumulated, fails to predict the LOB under basic assumptions. This is because if one item builds up a large lead the inhibition overwhelms the attention bias, meaning it is less important which item is being attended to. A model with feedforward inhibition, where inhibition acts at the level of input, performs much better. It can mimic a relative threshold model very closely when inhibition is 1, or at least very high. However, this breaks down quickly as the inhibition parameter is reduced to more behaviorally plausible levels.

The large scale grid search shows that both inhibition assumptions can predict the LOB under some parameters and can predict realistic RT distributions under others, but we find no overlap where either model can predict both simultaneously. The fact that the feed-forward model with complete inhibition is mathematically equivalent to the relative threshold models means that a grid search with sufficient resolution would find suitable parameters. However, our analyses have shown that the LOB predictions are very sensitive to any reduction in inhibition away from its maximum value of 1. Finding parameter sets for absolute threshold models at realistic levels of inhibition seems like a remote possibility. Furthermore, if such a set were found, the proportion of parameter space occupied must be small as it must fit in between the nodes of the grid we explored. Given the scale of variance seen in parameter estimates from behavioral data, it would be incredibly difficult to fit such a model to individuals’ responses.

## Figures and Tables

**Table 1 tbl1:** Summary of the Models Estimated Using Parameter Grid Search

Model	Decision criterion	Inhibition	Decay	Free parameters
Absolute threshold (independent race model)	Evidence for 1 or evidence for 2 is greater than threshold	NA	NA	2
Relative threshold	Difference between evidence for 1 and evidence for 2 is greater than threshold	NA	NA	2
Absolute threshold with feed forward inhibition	Evidence for 1 or evidence for 2 is greater than threshold	Input to each accumulator inhibits the input to competitors	Accumulated evidence decays as a proportion of total accumulated	4
Absolute threshold with mutual inhibition	Evidence for 1 or evidence for 2 is greater than threshold	Total evidence accumulated for an option inhibits the input to competitor	Accumulated evidence decays as a proportion of total accumulated	4

**Table 2 tbl2:** Criteria Used to Test Each Model’s Performance

Criteria	Tests behavioral or visual phenomena
The attention bias at the time of response is between .6 and .9	Visual
The average bias from the start of the choice until 2 seconds before response is between .45 and .55	Visual
The mean RT is between 2 and 8 seconds	Behavioral
The skewness of the RT distribution is positive and greater than 1	Behavioral
No more than 1% of choices where the model fails to make a response within 30 seconds	Behavioral

**Table 3 tbl3:** Proportion of Parameter Space Which Satisfies Different Numbers of Criteria for Each Model Type

Number of criteria satisfied	Relative threshold	Mutual inhibition	Feed forward inhibition
5	2%	0%	0%
4	27%	1%	1%
3	35%	6%	3%
2	26%	15%	16%
1	10%	61%	66%
0	0%	17%	14%

**Table 4 tbl4:** Proportion of Parameter Space Which Satisfies Each of the Five Model Criteria

Criteria	Relative threshold	Mutual inhibition	Feed forward inhibition
End bias .6–.9	20%	4%	3%
Early stage bias .45–.55	85%	21%	23%
Mean RT 2–8s	35%	21%	15%
Positive skew in RT distribution	77%	18%	12%
99% of decisions made in 30s	66%	48%	57%

**Figure 1 fig1:**
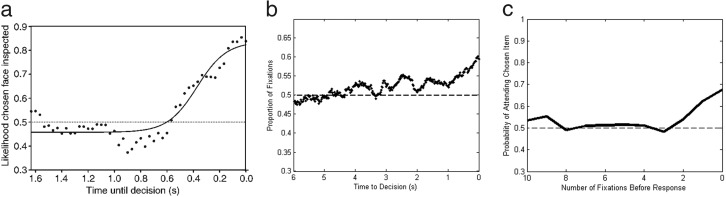
Empirically measured late onset bias during (a) preferential binary choice between faces (Shimojo et al., 2003); (b) multiattribute choice between 2 apartments each with 5 numerical attributes (Mullett and Tunney, 2016), and (c) choices between two risky gambles (Stewart, Hermans, & Matthews, 2015). Note that whereas a and b are plotted against time, c is plotted by discrete fixations.

## Appendix A Distribution of Fixation Durations Sampled for Simulations

[Fig-anchor fig17]

## B Effect of Minor Changes to Inhibition Parameter in a Feed-Forward Model

[Fig-anchor fig18]
