# Correction to Mullet and Stewart (2016)

**DOI:** 10.1037/dec0000062

**Published:** 2016-05-23

In the article “Implications of Visual Attention Phenomena for Models of Preferential Choice,” by Timothy L. Mullett and Neil Stewart (*Decision*, Advance online publication. February 1, 2016. http://dx.doi.org/10.1037/dec0000049), the copyright should have been “© 2016 The Author(s)”. The author note also should have included the following license statement “This article has been published under the terms of the Creative Commons Attribution License (http://creativecommons.org/licenses/by/3.0/), which permits unrestricted use, distribution, and reproduction in any medium, provided the original author and source are credited. Copyright for this article is retained by the author(s). Author(s) grant(s) the American Psychological Association the exclusive right to publish the article and identify itself as the original publisher.” The online version of this article has been corrected.

